# Supplementary material for: Hilar mossy cells control structural and functional organization in the dentate gyrus
Source: bioRxiv. 2026 Jul 17:2026.07.15.738260. Preprint. [Version 1] doi: 10.64898/2026.07.15.738260 (PMC13405028; doi:10.64898/2026.07.15.738260)
Supplement: 1 [file NIHPP2026.07.15.738260v1-supplement-1.pdf]

bioRxiv preprint doi: https://doi.org/10.64898/2026.07.15.738260; this version posted July 17, 2026. The copyright holder for this preprint (which was not certified by peer review) is the author/funder. This article is a US Government work. It is not subject to copyright under 17 USC 105 and is also made available for use under a CC0 license.

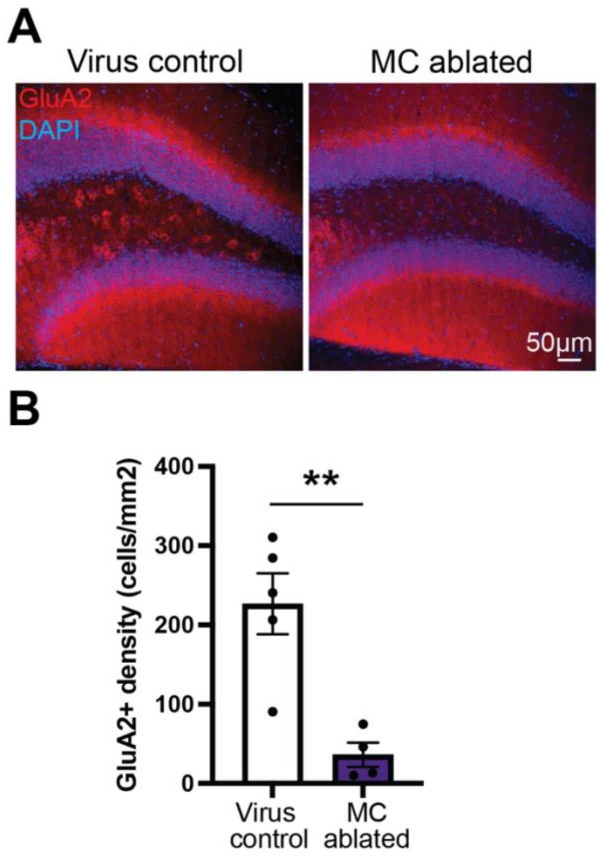

Supplemental Figure 1: Mossy cell ablation by AAV5-flex-taCasp3 in dorsal hippocampus. A) Representative sections from dorsal hippocampus from virus control and mossy cell (MC) ablated mice, 6 weeks after AAV5-flex-taCasp3 virus injection. Tissue was stained for glutamate receptor AMPA type subunit 2 (GluA2, red) and co-stained for DAPI (blue) to visualize the dentate granule cell layer and CA3 pyramidal cell layer, which also express GluA2. B) GluA2+ hilar cell density is reduced in MC ablated (N=4 mice) mice compared to virus control (N=5 mice) mice (** $p>0.01$).

bioRxiv preprint doi: https://doi.org/10.64898/2026.07.15.738260; this version posted July 17, 2026. The copyright holder for this preprint (which was not certified by peer review) is the author/funder. This article is a US Government work. It is not subject to copyright under 17 USC 105 and is also made available for use under a CC0 license.

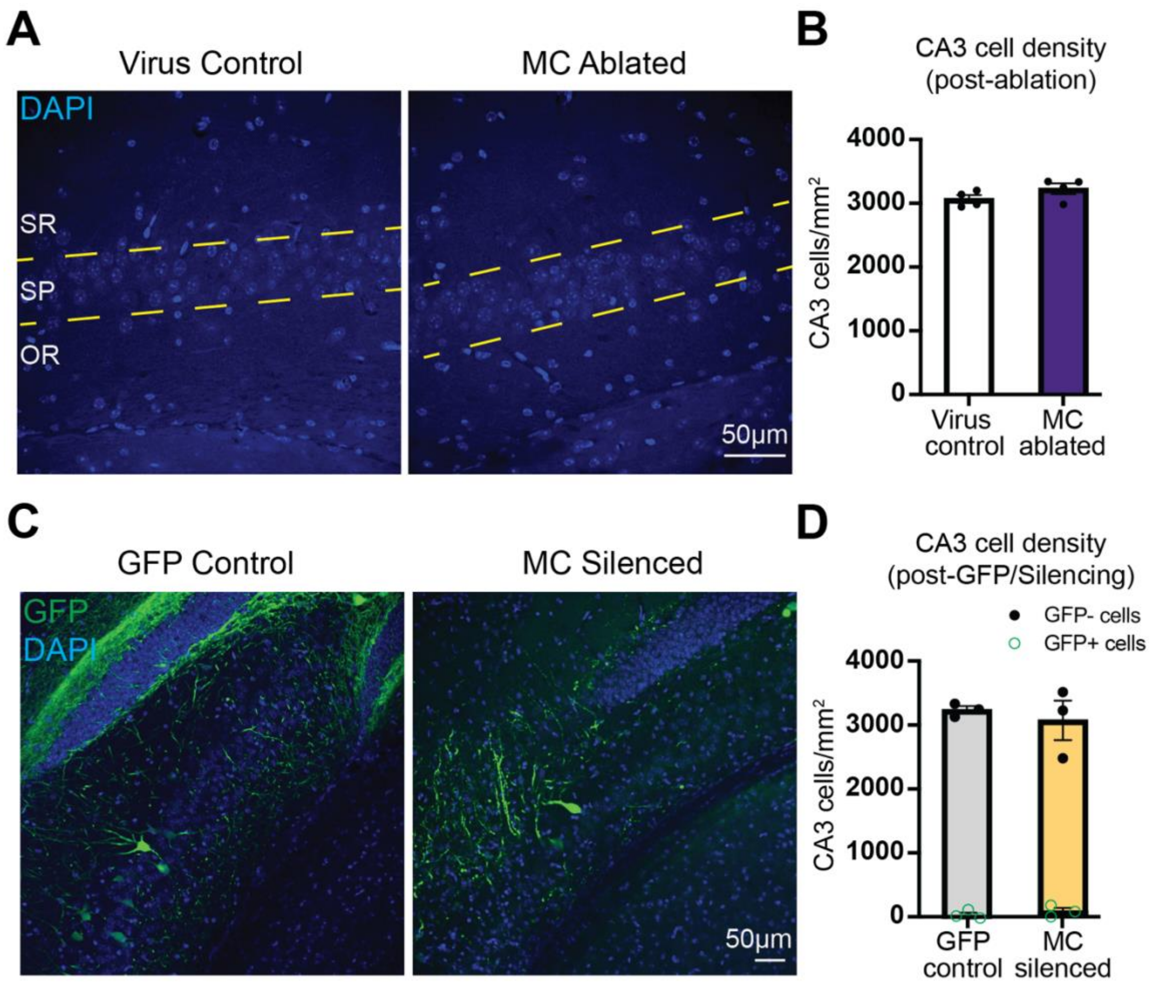

Supplemental Figure 2: Minimal off-target viral expression in CA3 neurons. A) Representative images of DAPI-stained pyramidal cell nuclei in proximal CA3 from virus control and mossy cell (MC) ablated mice. Yellow dashed lines represent borders of the pyramidal cell layer (SP), stratum oriens (OR) and stratum radiatum (SR). B) CA3 cell densities were similar between virus control (N= 4 mice) and MC ablated conditions (N= 4 mice), suggesting minimal ablation of CA3 pyramidal neurons following AAV5-flex-taCasp3 virus injection (p=0.17). C) Representative images of GFP-expressing neurons in CA3 from Crlr-Cre mice injected with AAV5-flex-GFP or AAV5-flex-TeLC-GFP viruses, co-stained with DAPI. D) Cell densities of both GFP-negative (GFP control, N= 3 mice; MC Silenced, N= 3 mice; p=0.60) and GFP-positive (p=0.57) were not different between experimental groups.

bioRxiv preprint doi: https://doi.org/10.64898/2026.07.15.738260; this version posted July 17, 2026. The copyright holder for this preprint (which was not certified by peer review) is the author/funder. This article is a US Government work. It is not subject to copyright under 17 USC 105 and is also made available for use under a CC0 license.

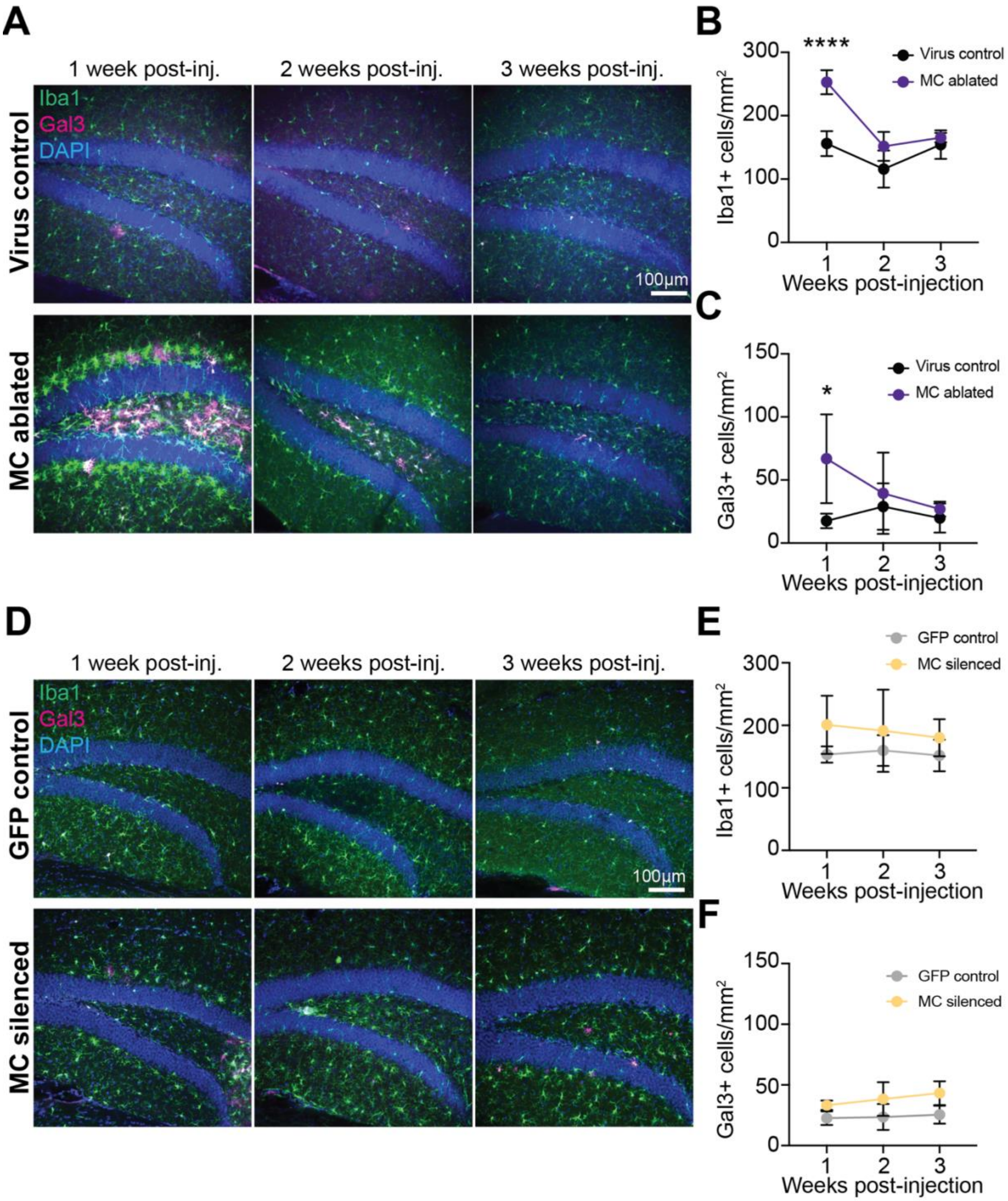

Supplemental Figure 3: Timecourse of microglial activation after mossy cell ablation. A) Representative dentate sections 1, 2, or 3 weeks following AAV flex Casp3 injections into Crlr-Cre-negative (Virus control) or Crlr-Cre-positive (MC ablated) mice.

bioRxiv preprint doi: https://doi.org/10.64898/2026.07.15.738260; this version posted July 17, 2026. The copyright holder for this preprint (which was not certified by peer review) is the author/funder. This article is a US Government work. It is not subject to copyright under 17 USC 105 and is also made available for use under a CC0 license.

Microglia are stained with anti-Iba1 (green), with activated microglia identified by anti-Gal3 (red) staining. B) Iba1-positive cell densities at different timepoints after virus injection; (Virus control n = 4 mice at 1 week post-virus, 4 mice at 2 weeks, and 4 mice at 3 weeks; MC ablated n = 6 mice at 1 week, 5 mice at 2 weeks, and 4 mice at 3 weeks (**** $p < 0.0001$ at 1 week; n.s. at 2 and 3 weeks). C) Gal3-positive cell densities at different timepoints after virus injection (* $p < 0.05$ at 1 week; n.s. at 2 and 3 weeks). D) Representative dentate sections 1, 2, and 3 weeks following AAV flex GFP (GFP control) or AAV flex TeLC-GFP (MC silenced) injections into Crlr-Cre-positive mice. Microglia are stained with anti-Iba1 (green, pseudocolored), with activated microglia identified using anti-Gal3 (red) staining. E) Iba1-positive cell densities at different timepoints after virus injection (GFP control n = 3 mice at 1 week post-virus, 3 mice at 2 weeks, and 4 mice at 3 weeks; MC silenced= 3 mice at 1 week, 3 mice at 2 weeks, and 3 mice at 3 weeks (n.s. at all timepoints). F) Gal3-positive cell densities at different timepoints after control virus injection or MC silencing (n.s. at all timepoints).

bioRxiv preprint doi: https://doi.org/10.64898/2026.07.15.738260; this version posted July 17, 2026. The copyright holder for this preprint (which was not certified by peer review) is the author/funder. This article is a US Government work. It is not subject to copyright under 17 USC 105 and is also made available for use under a CC0 license.

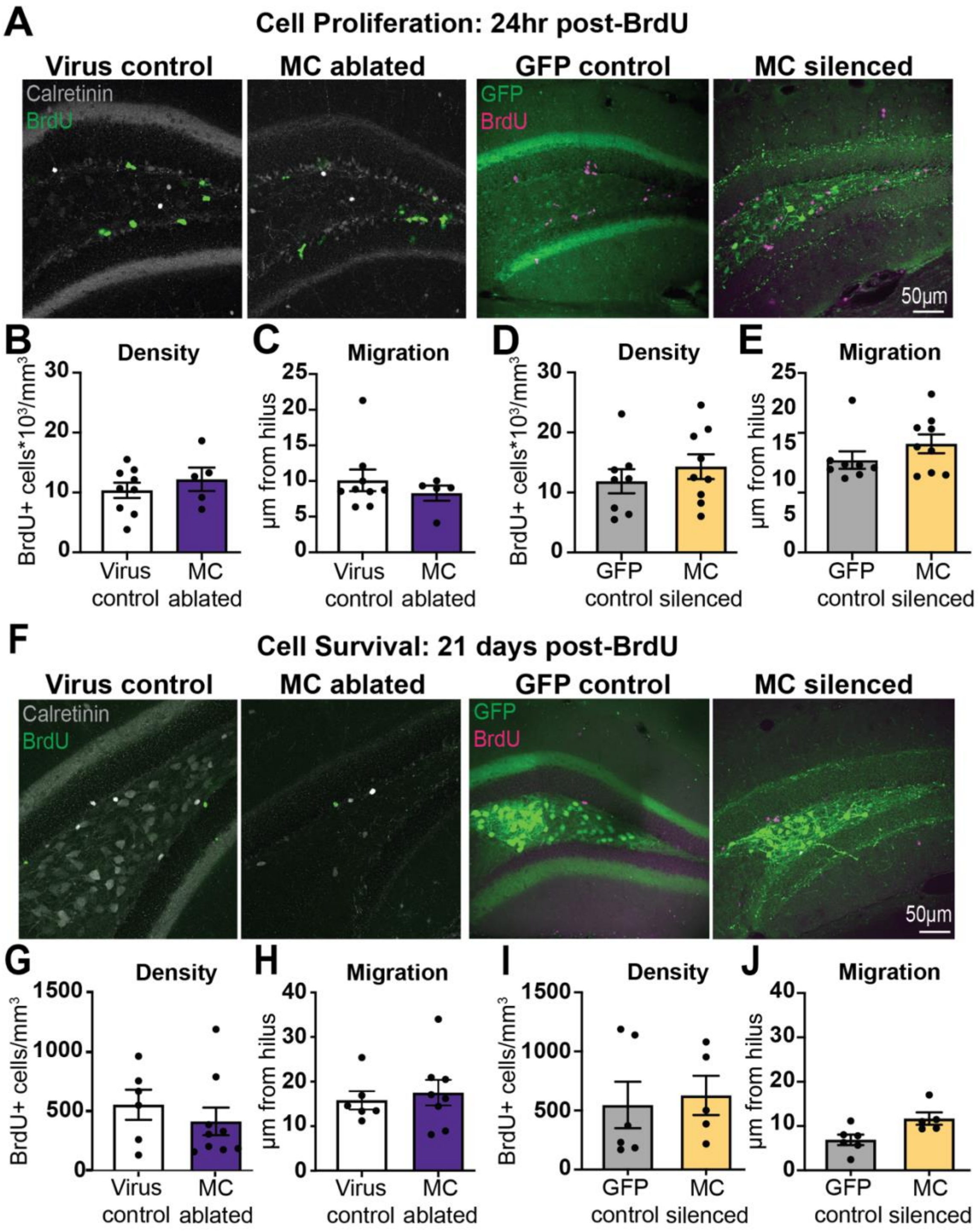

Supplemental Figure 4: Cell proliferation and survival in the dentate granule cell layer are not altered following mossy cell manipulations. A) Representative mitotic labeling in virus controls, MC ablated, and MC silenced conditions 24hr after BrdU administration to assess cell proliferation. B&C) Cell proliferation and outward migration

bioRxiv preprint doi: https://doi.org/10.64898/2026.07.15.738260; this version posted July 17, 2026. The copyright holder for this preprint (which was not certified by peer review) is the author/funder. This article is a US Government work. It is not subject to copyright under 17 USC 105 and is also made available for use under a CC0 license.

in virus control and MC ablated conditions (Virus control n = 9 mice, MC ablated n = 5 mice; n.s. both measures). D&E) Cell proliferation and outward migration in GFP control and MC silenced conditions (GFP control n = 8 mice, MC silenced n = 9 mice; n.s. both measures). F) Representative BrdU labeling in virus controls, MC ablated, and MC silenced conditions 21 days after BrdU administration to assess newborn cell survival. G&H) BrdU-positive cell density and outward migration distance in virus control and MC ablated conditions, 21 days after BrdU administration (Virus control n = 6 mice, MC ablated n = 9 mice; n.s. both measures). I&J) BrdU-positive cell density and outward migration distance in GFP control and MC silenced conditions, 21 days after BrdU administration (GFP control n = 6 mice, MC silenced n = 5 mice; n.s. both measures).

bioRxiv preprint doi: https://doi.org/10.64898/2026.07.15.738260; this version posted July 17, 2026. The copyright holder for this preprint (which was not certified by peer review) is the author/funder. This article is a US Government work. It is not subject to copyright under 17 USC 105 and is also made available for use under a CC0 license.

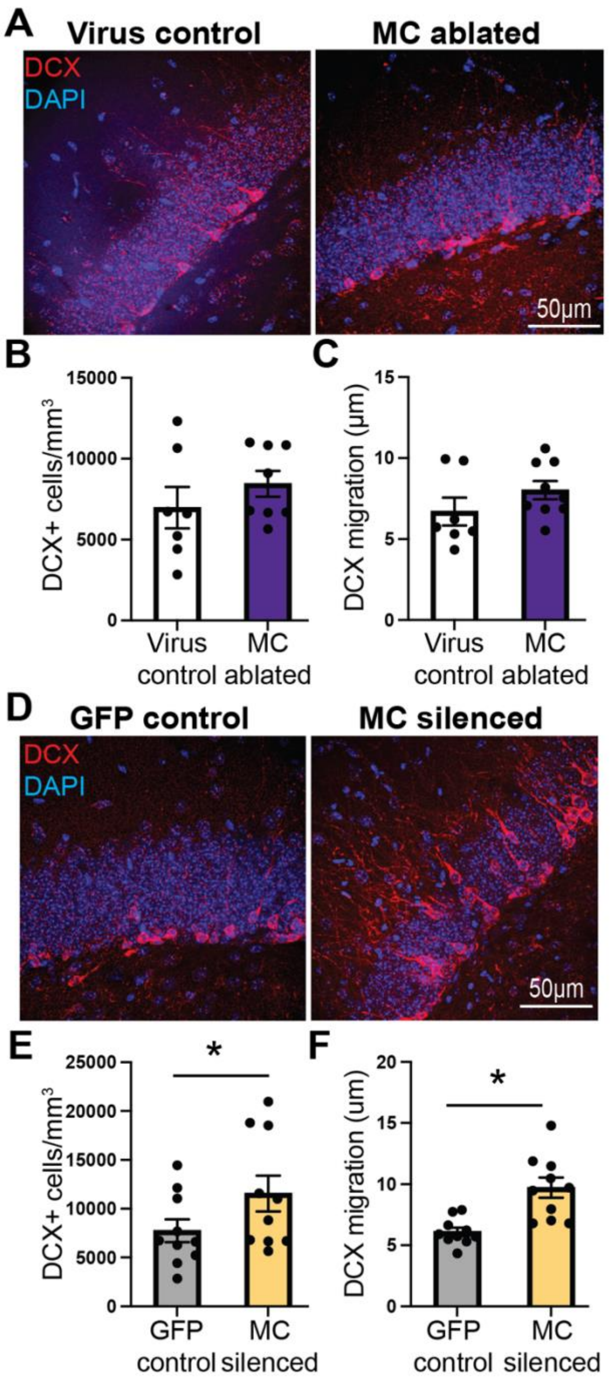

Supplemental Figure 5: Differential effects of mossy cell ablation vs silencing on immature granule cells. A) Representative immature adult-born granule cells, as assessed by doublecortin (DCX) staining (red) in virus control and MC ablated mice. B&C) DCX-positive cell density and outward migration from the hilus, demonstrating no change following mossy cell ablation (Virus control n = 7 mice, MC ablated n = 8 mice;

bioRxiv preprint doi: https://doi.org/10.64898/2026.07.15.738260; this version posted July 17, 2026. The copyright holder for this preprint
(which was not certified by peer review) is the author/funder. This article is a US Government work. It is not subject to copyright under 17 USC
105 and is also made available for use under a CC0 license.

n.s. both measures). D) Representative immature adult-born granule cells (DCX+; red) in GFP control and MC silenced mice. E&F) DCX staining demonstrates a mildly increased density of immature granule cells and increased outward migration of immature cells following mossy cell silencing (GFP control n = 10 mice, MC silenced n = 10 mice; * = p <0.05).

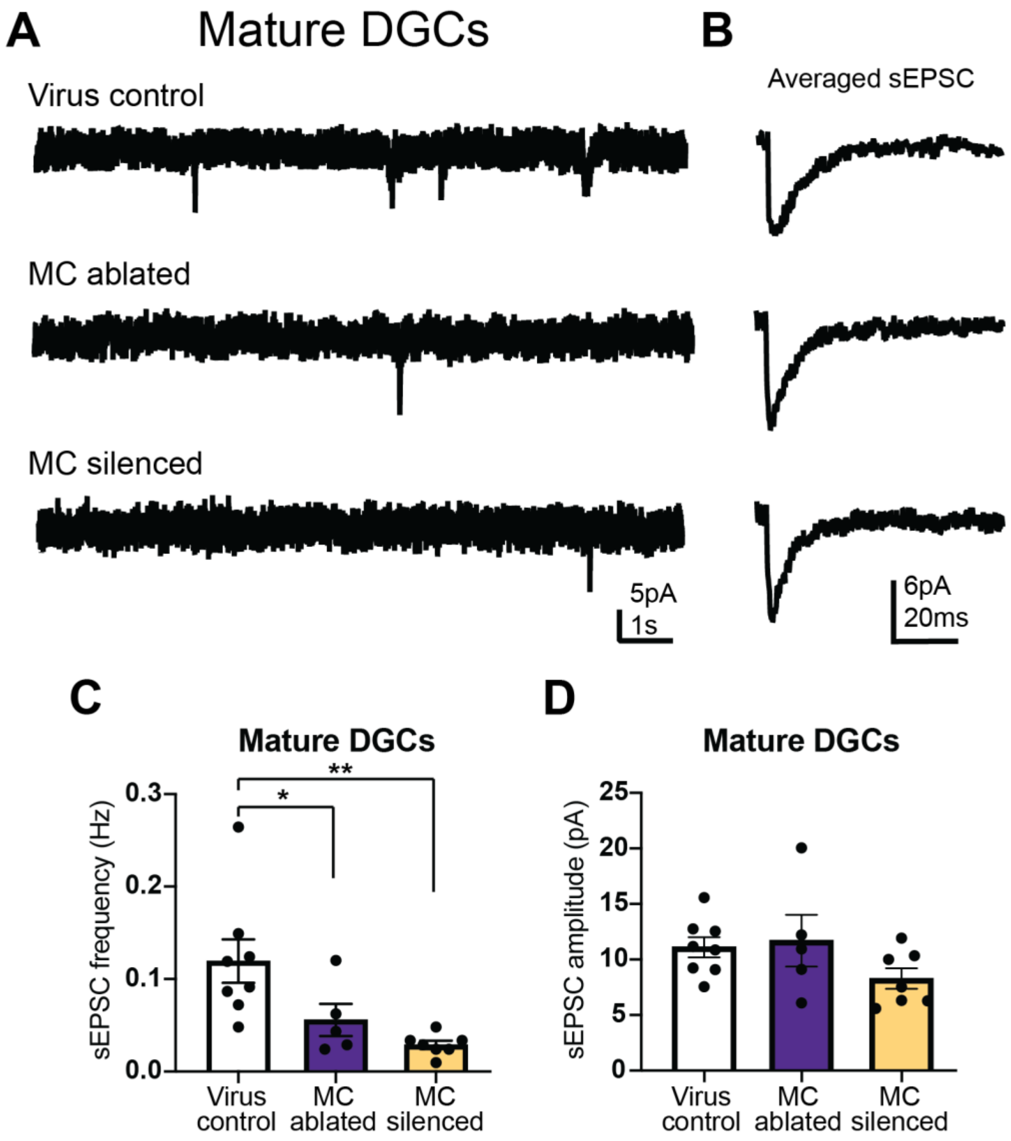

Supplemental Figure 6: Reduced spontaneous excitatory synaptic currents in mature DGCs after mossy cell ablation or silencing. A) Representative spontaneous

bioRxiv preprint doi: https://doi.org/10.64898/2026.07.15.738260; this version posted July 17, 2026. The copyright holder for this preprint (which was not certified by peer review) is the author/funder. This article is a US Government work. It is not subject to copyright under 17 USC 105 and is also made available for use under a CC0 license.

excitatory post-synaptic current (sEPSC) recordings from mature DGCs from virus control, MC ablated, and MC silenced mice. B) Average sEPSC waveforms from the respective recordings in A. C&D) sEPSC frequencies and amplitudes recorded from mature DGCs from virus control, MC ablated, and MC silenced mice. (sEPSC frequency * = $p < 0.05$, ** $p < 0.01$; sEPSC amplitude n.s. all comparisons).

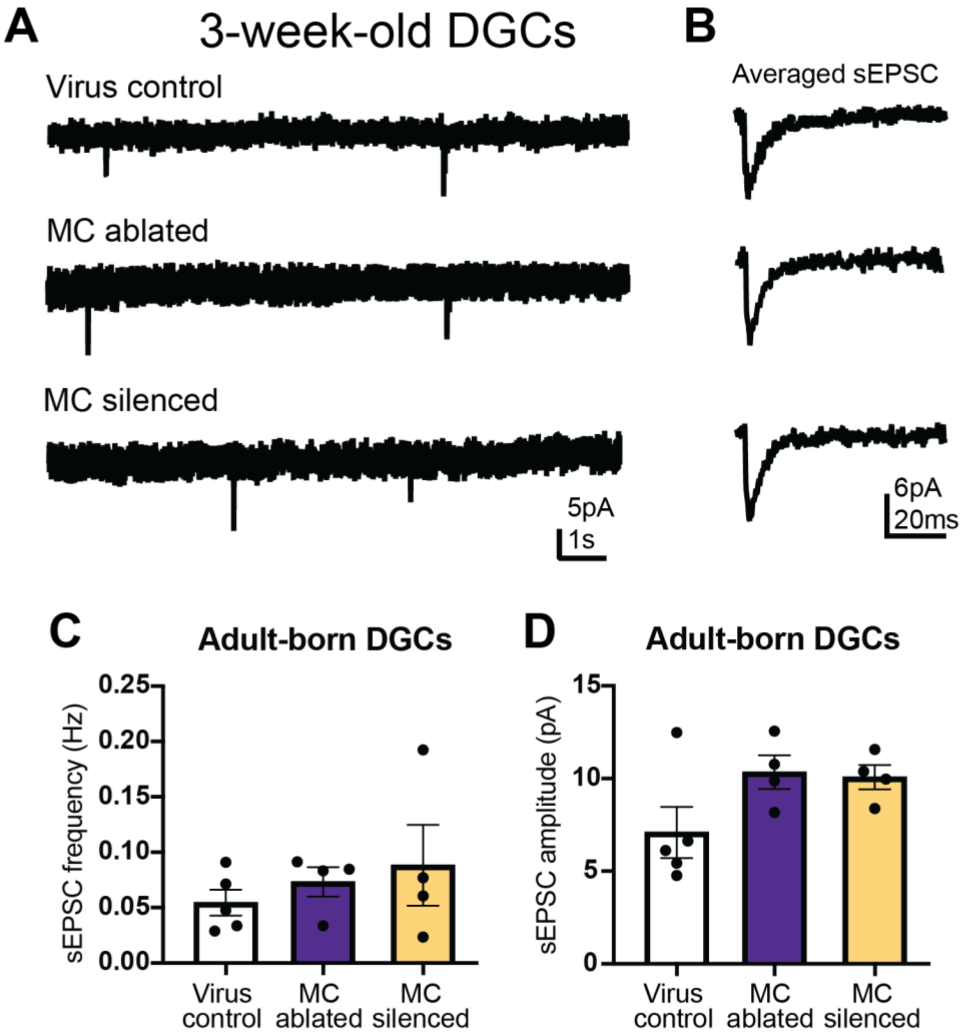

Supplemental Figure 7: sEPSCs are unchanged in immature DGCs generated after mossy cell ablation or silencing. A) Representative sEPSC recordings from 21 day-old adult-born DGCs from virus control, MC ablated, and MC silenced mice. B) Average sEPSC waveforms from the respective recordings in A. C&D) sEPSC

bioRxiv preprint doi: https://doi.org/10.64898/2026.07.15.738260; this version posted July 17, 2026. The copyright holder for this preprint (which was not certified by peer review) is the author/funder. This article is a US Government work. It is not subject to copyright under 17 USC 105 and is also made available for use under a CC0 license.

frequencies and amplitudes in 21 day old DGCs from virus control, MC ablated, and MC silenced mice. Neither sEPSC frequency or amplitude was different in adult-born cells in each group. (n.s. all comparisons).

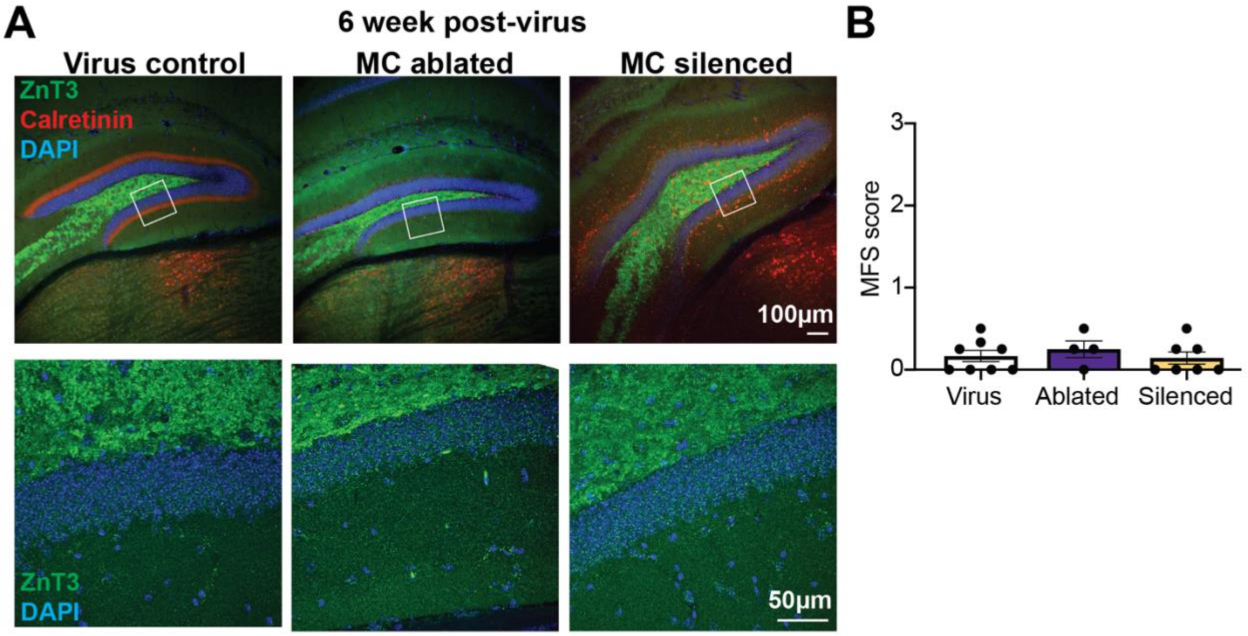

Supplemental Figure 8: Neither mossy cell ablation or silencing drive recurrent granule cell axon (mossy fiber) sprouting. A) Representative 5X (top) and 40X images (bottom) of mossy cell axon (calretinin, red) and granule cell mossy fiber bouton (ZnT3, green) staining in virus control, MC ablated, and MC silenced conditions 6 weeks after virus injection. B) Mossy fiber sprouting (MFS) is not detected in either MC ablated or MC silenced groups (Virus control n = 8 mice, MC ablated n = 4 mice, MC silenced n = 8 mice; n.s. all groups).

bioRxiv preprint doi: https://doi.org/10.64898/2026.07.15.738260; this version posted July 17, 2026. The copyright holder for this preprint (which was not certified by peer review) is the author/funder. This article is a US Government work. It is not subject to copyright under 17 USC 105 and is also made available for use under a CC0 license.

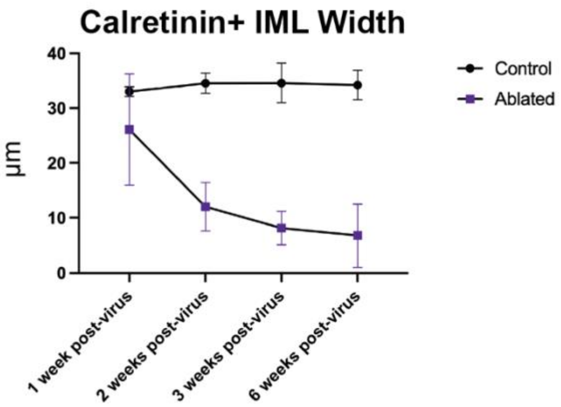

Supplemental Figure 9: Calretinin-positive IML width decreases during the first 2 weeks after mossy cell ablation and remains stable at later time points.
